# 

**Published:** 1887-06-18

**Authors:** 


					A*' ({Ml '>1*1
PRICE ONE PENNY.
No. 38, Vol. II.
June 18th, 1887.
I) \WHi /1H1 .
Monthly Parts, 6d.; post free, 7id.
"as a Sewspaper ] P?St ?fflCe ^ ^ Ditto per Ann., 7s. 6d., post free.
*.
ait Institution, .-Jfanttlg, antr ^ $atocf)taI Journal of
j^ospttals, &st>Iunts. & all ^genctcg for tfte (Kate of tfte g>tcfe, (grtticfem & $ctos.
HOSPITAL SUNDAY.
SECOND SPECIAL NUMBER.

				

